# Intestinal perforation secondary to ingested chicken bone: case report and literature review

**DOI:** 10.3389/fsurg.2026.1817632

**Published:** 2026-05-15

**Authors:** Danning Zhang, Zining Chen, Xiaolong He, Ming Xie, Qingbo Feng

**Affiliations:** 1 Zunyi Medical University, Zunyi, Guizhou, China; 2 Affiliated Hospital of Zunyi Medical University, Zunyi, Guizhou, China; 3 Department of General Surgery, Digestive Disease Hospital, Affiliated Hospital of Zunyi Medical University, Zunyi, Guizhou, China; 4 Guizhou Provincial Key Laboratory of Digestive System Diseases, Zunyi City, Guizhou Province, China

**Keywords:** case report, chicken bone, foreign body ingestion, intestinal perforation, laparoscopy

## Abstract

Foreign body ingestion is usual in daily life, particularly prevalent in the pediatric population and the elderly, but it can also occur in adults due to eating quickly or inadequate mastication. Most foreign bodies can pass through the gastrointestinal tract spontaneously without intervention. However, hard and sharp foreign bodies may penetrate through gastric and duodenal walls. Small bowel perforation caused by chicken bones is rare in clinical practice. It presents no specific symptoms and causing diagnosis delayed. Endoscopy is limited for foreign bodies in the lower gastrointestinal tract, while abdominal computed tomography (CT) plays a key role in detecting radiopaque foreign bodies and related complications. In terms of treatment, laparoscopic surgery has become an optimal choice for foreign body extraction given its advantages of minimal trauma, clear visualization, and rapid postoperative recovery. Here, we report the diagnosis and management of a 28-year-old male with a acute small bowel perforation caused by an ingested chicken bone fragment. The patient initially presented with oropharyngeal discomfort during chicken consumption, but initial laryngoscopic evaluation revealed no abnormalities. Within hours, he developed progressive, non-resolving abdominal pain, prompting further investigation with abdominal computed tomography (CT), which identified a hyperdense linear object within the distal ileum, consistent with foreign body perforation. The patient underwent successful laparoscopic extraction of the chicken bone fragment with primary repair of the perforation site, experiencing an uneventful recovery and discharge on postoperative day 7.

## 1 Introduction

Foreign body ingestion is a common condition that can affect individuals of all ages. The incidence is relatively high among children, the elderly, and those with dysphagia or mental disorders (1). Most foreign bodies can spontaneously pass through the gastrointestinal tract without any intervention. However, hard and sharp foreign bodies may penetrate the gastric and duodenal walls. It may be related to the thinner walls of the stomach and duodenum or the longer time for foreign bodies stay in these segments (2, 3). Due to anatomical distance, these foreign bodies are easy to pass through the hole and migrate to the adjacent organs, such as pancreas, liver, and appendix, leading to complications such as pancreatitis, liver abscess and appendicitis (4–6).

Common ingested foreign bodies include animal bones, sewing needles, toothpicks, etc. It's notable that many patients swallow foreign bodies unintentionly. The internal between the onset of clinical manifestations and foreign body ingestion may range from one week to more than one year (3, 7). Compared to gastric or colonic perforation, small intestinal perforation caused by chicken bone fragments is rare in clinical practice. Endoscopic retrieval becomes limited once the foreign body has passed into the lower gastrointestinal tract. In such cases, abdominal computed tomography (CT) is crucial for detecting foreign bodies and complications (2, 8, 9).

Traditional open surgery once was the standard treatment for perforation. However,laparoscopic surgery has now become the preferred option due to its advantages, including less trauma, clearer surgical vision, faster postoperative recovery, and shorter hospital stay (10–12). Here, we present the diagnosis and management of a 28-year-old male with an acute small bowel perforation caused by an ingested chicken bone fragment.

## 2 Case presentation

### 2.1 Patient history

A 28-year-old Chinese male with no significant past medical history (no hypertension, diabetes mellitus, or prior abdominal surgery) presented to the emergency department of our hospital with acute abdominal pain. The patient reported accidental ingestion of a small chicken bone 2 days prior to admission while consuming roast chicken, without thorough chewing. Immediately after ingestion, he experienced transient mild throat discomfort but no chest pain, dysphagia, or abdominal pain. He subsequently visited a local otolaryngology clinic where flexible laryngoscopy was performed, revealing no foreign body in the pharynx or larynx and normal mucosal surfaces. The patient was advised to observe at home, maintain a soft diet, and seek urgent medical attention if abdominal pain, fever, vomiting, or other concerning symptoms developed.

Approximately 36 h post-ingestion, the patient developed persistent dull abdominal pain, initially diffuse but gradually localizing to the right lower quadrant. The pain worsened with movement, coughing, or deep breathing. He denied nausea, vomiting, diarrhea, constipation, rectal bleeding, or fever, with no recent weight loss or changes in appetite. His last bowel movement was 1 day prior to admission, which was normal in consistency and color.

### 2.2 Physical examination

On admission, the patient's vital signs were stable: temperature 37.2 °C, heart rate 88 beats per minute, respiratory rate 18 breaths per minute, and blood pressure 125/80 mmHg. General physical examination revealed an alert, oriented male in mild distress due to abdominal pain. The skin was warm and dry without cyanosis or jaundice, and no lymphadenopathy was palpable in the neck, axillae, or groin. Cardiopulmonary examination was unremarkable, with clear bilateral breath sounds and no cardiac murmurs.

Abdominal examination showed a flat abdomen with normal bowel sounds (approximately 4 per minute). Tenderness was elicited in the right lower quadrant, particularly at McBurney's point, without rebound tenderness or guarding. The abdomen was soft, with no palpable masses. Percussion revealed no dullness or shifting dullness. Rectal examination was unremarkable, with no tenderness or masses, and the stool guaiac test was negative.

### 2.3 Laboratory investigations

Admission laboratory investigations included complete blood count, basic metabolic panel, liver function tests, and inflammatory markers, with results as follows: white blood cell count (WBC) 11.5 × 10⁹/L (normal range: 4.0–10.0 × 10⁹/L), neutrophil percentage 78% (normal range: 50%–70%), lymphocyte percentage 18% (normal range: 20%–40%), hemoglobin 145 g/L (normal range: 120–160 g/L), platelet count 230 × 10⁹/L (normal range: 100–300 × 10⁹/L), C-reactive protein (CRP) 8 mg/L (normal range: 0–10 mg/L), blood urea nitrogen (BUN) 5.2 mmol/L (normal range: 3.2–7.1 mmol/L), and creatinine 88 μmol/L (normal range: 53–106 μmol/L). Electrolytes (sodium, potassium, chloride) and liver function tests (alanine transaminase, aspartate transaminase, total bilirubin, alkaline phosphatase) were all within normal ranges.

### 2.4 Imaging studies

**Figure 1:**
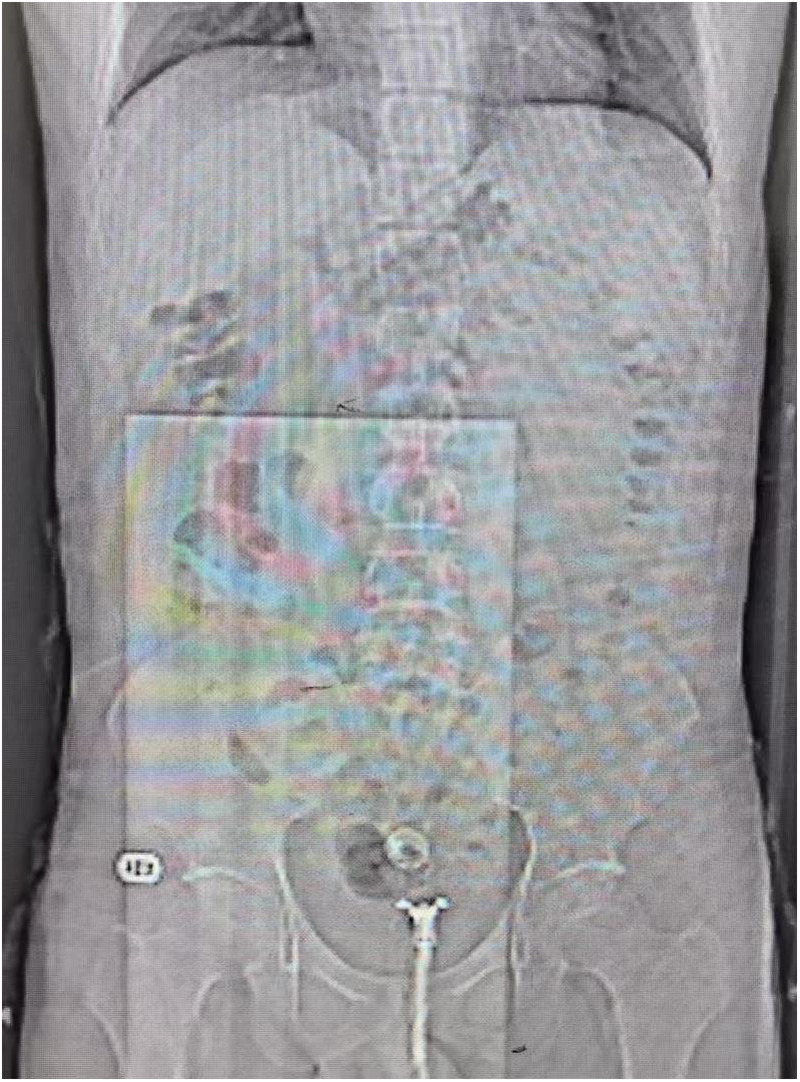
x-ray was initially performed but failed to clearly identify the foreign body or signs of perforation.

An emergency abdominal x-ray was initially performed but failed to clearly identify the foreign body or signs of perforation (e.g., free air under the diaphragm) (Figure 1). Given the patient's clinical symptoms, history of chicken bone ingestion, and elevated WBC count, an abdominal contrast-enhanced CT scan was performed for further evaluation.

**Figure 2:**
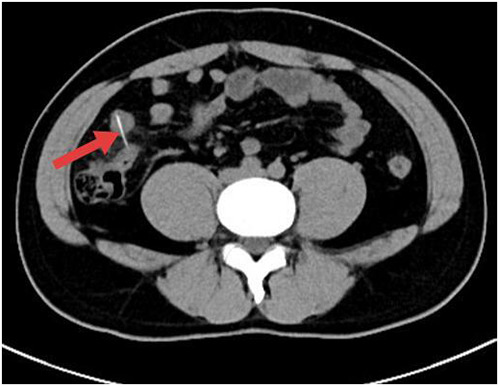
CT scan showing a linear hyperdense foreign body (red arrow) in the right lower quadrant.

The CT scan demonstrated a linear hyperdense structure measuring approximately 2.0 cm × 0.4 cm in the right lower quadrant, adjacent to the terminal ileum (Figure 2), consistent with a chicken bone. Additionally, localized fat stranding, mild peritoneal thickening around the terminal ileum, and a small amount of free peritoneal fluid were noted, consistent with localized peritonitis secondary to small intestinal perforation. No large abscess formation, intestinal obstruction, or involvement of other abdominal organs was identified.

### 2.5 Preoperative diagnosis

Based on the patient's history of chicken bone ingestion, clinical manifestations of right lower quadrant abdominal pain, physical examination findings of right lower quadrant tenderness, elevated WBC count, and CT scan findings of a linear hyperdense foreign body adjacent to the terminal ileum with signs of localized peritonitis, the preoperative diagnosis was established as small intestinal perforation caused by an ingested chicken bone.

### 2.6 Surgical intervention

Emergency laparoscopic exploration was planned following informed consent. The patient was kept nil per os (NPO) preoperatively, with intravenous fluid resuscitation, prophylactic antibiotics (cefuroxime 1.5 g and metronidazole 500 mg), and analgesics administered.

The procedure was performed under general anesthesia with endotracheal intubation. The patient was placed in a supine position with legs apart. A 10 mm trocar was inserted at the umbilicus using the open Hasson technique, and pneumoperitoneum was established with carbon dioxide at an intra-abdominal pressure of 12–14 mmHg. A 10 mm laparoscope was inserted via the umbilical trocar for initial abdominal exploration, with two additional 5 mm trocars placed in the left lower quadrant and right upper quadrant as working ports.

**Figure 3:**
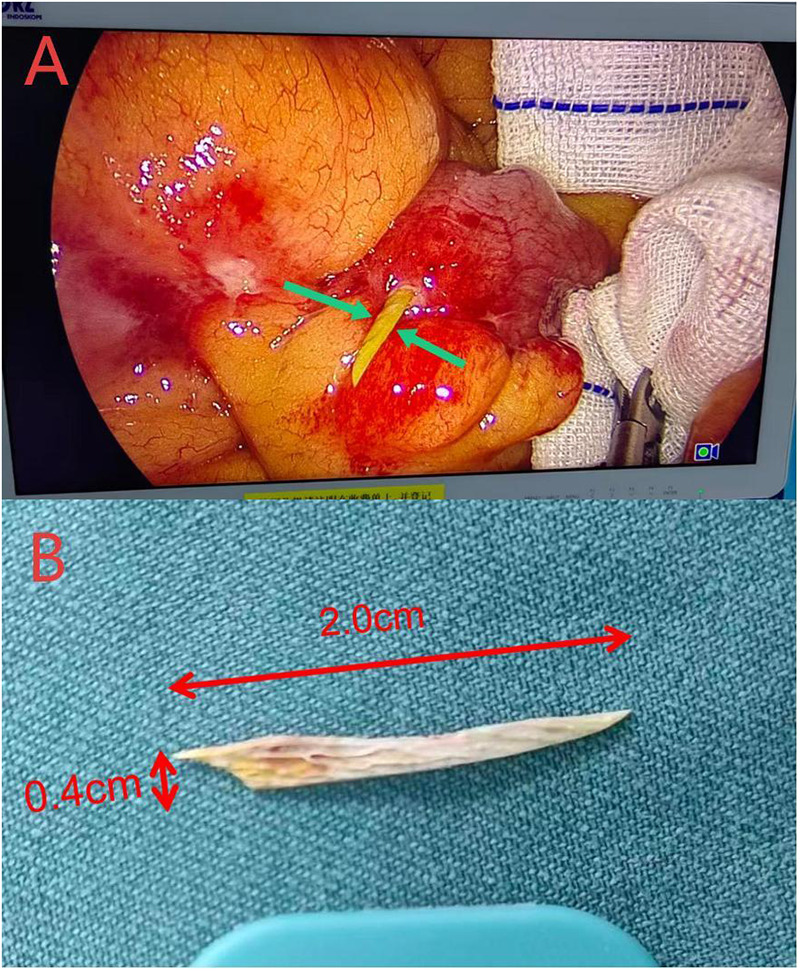
Intraoperative laparoscopic findings. **(A)** A chicken bone (green arrow) penetrating the antimesenteric border of the terminal ileum; **(B)** The extracted chicken bone.

Intraoperative exploration revealed a small amount of turbid peritoneal fluid in the pelvic cavity and right lower quadrant. Severe adhesions were identified between the terminal ileum (approximately 15 cm proximal to the ileocecal valve) and surrounding omentum and peritoneal tissues. These adhesions were carefully dissected using an ultrasonic scalpel to expose the terminal ileum, revealing a 2.0 cm × 0.4 cm chicken bone penetrating the antimesenteric border of the terminal ileum, with its sharp end protruding into the peritoneal cavity (Figures 3A, B). The surrounding intestinal mucosa was congested and edematous, with a small amount of purulent exudate around the 0.5 cm diameter perforation site. No other intestinal lesions or foreign bodies were detected during the exploration.

Under direct laparoscopic visualization, the chicken bone was gently grasped with atraumatic forceps and carefully extracted to avoid further intestinal mucosal injury. The perforation site was inspected to ensure no residual foreign body fragments remained, then repaired with interrupted sutures using 3-0 absorbable polyglycolic acid sutures, with careful approximation of the intestinal mucosa without excessive tension. Post-repair, the suture line was tested for leakage by gentle air insufflation via a nasogastric tube, with no air leakage detected.

The abdominal cavity was thoroughly irrigated with 3,000 mL of warm normal saline to remove purulent exudate, foreign body debris, and inflammatory mediators. A drainage tube was placed in the right lower quadrant near the repaired perforation site. Trocar sites were closed in layers: the umbilical port fascia with 2-0 absorbable sutures, and skin incisions at all ports with subcutaneous sutures, covered with sterile dressings.

Total operative time was 85 min, with an estimated blood loss of approximately 30 mL. The patient tolerated the procedure well without intraoperative complications.

### 2.7 Postoperative management and outcomes

Postoperatively, the patient was transferred to the surgical ward for monitoring. He was maintained on NPO status initially, with continued intravenous fluid resuscitation, antibiotics (cefuroxime 1.5 g every 8 h and metronidazole 500 mg every 12 h) for 48 h, and analgesics as needed. The nasogastric tube was removed on postoperative day 1, and clear liquids were initiated on postoperative day 2, which was well-tolerated. The patient gradually advanced to a soft diet on postoperative day 3 and a regular diet on postoperative day 5 without gastrointestinal symptoms (e.g., nausea, vomiting, abdominal pain).

Drainage tube output was minimal (<50 mL/day) and gradually decreased, with tube removal on postoperative day 4. Postoperative day 3 laboratory tests showed a normal WBC count (7.8 × 10⁹/L) and CRP level (3 mg/L). The patient's recovery was uneventful, with no signs of infection, intestinal obstruction, or anastomotic leakage. He was discharged on postoperative day 7 with instructions to maintain a soft diet for 2 weeks, avoid strenuous physical activity for 1 month, and attend a 1-month outpatient follow-up.

At the 1-month follow-up, the patient reported no abdominal pain, nausea, vomiting, or other discomfort. Physical examination revealed well-healed surgical incisions, no abdominal tenderness or masses, and normal bowel sounds, with normal complete blood count and inflammatory markers on laboratory testing. At the 3-month follow-up, the patient remained asymptomatic, had resumed normal daily activities and work, and abdominal ultrasound showed no abdominal abnormalities, including no evidence of intestinal obstruction or abscess formation.

## 3 Literature review

We reviewed reports of jejunoileal perforation caused by foreign body ingestion in the past three years (Table 1). Patients were predominantly male (only 2 female cases) with a broad age range (2–75 years). Sharp rigid foreign bodies, such as toothpicks, fish bones, chicken bones, grill brush bristles, were predominant. There are also special types such as magnets and plant stems been reported. Toothpicks (3 cases) and fish bones (4 cases) were the most common causes. Most of the foreign bodies measured between 2.3 cm and 5 cm in size. CT was the most common imaging modality (12 cases), followed by x-ray (9 cases), with ultrasound used rarely (1 case). Both open and laparoscopic surgery were employed, with laparoscopic use rising markedly in recent years (only about 17% cases in 2023 was treated with laparoscopic surgery, compared with 80% cases in 2025). Laparoscopic cases had shorter hospital stays (average 5.29 days) compared to open surgery (average 10 days, maximum 27 days).

**Table 1:** clinical data of jejunoileal perforation caused by foreign body ingestion.

Year	Sex	Age	Foreign body	Size (cm)	Perforation site	Imaging examination	Surgical approach	Hospital stay (days)	Ref.
2023	M	8	Toothpick	NA	Ileum	x-ray	Open surgery	NA	(13)
2023	M	48	Toothpick	NA	Ileum	CT	Open surgery	12	(14)
2023	M	45	Right-angled thorns of vachellia nilotica	5	Ileum	x-ray, CT	Open surgery	8	(15)
2023	M	NA	Magnet	NA	Jejunum, cecum	x-ray, CT	Open surgery	4	(16)
2023	M	65	Bread tag	NA	Jejunum	CT	Laparoscopy	5	(17)
2023	M	63	Fish bone	NA	Jejunum	x-ray, CT	Open surgery	6	(18)
2024	M	60	Chicken bone	4	Ileum	x-ray	Open surgery	8	(19)
2024	M	65	Coconut leaf midrib skewer	3	Ileum	x-ray	Open surgery	5	(20)
2024	M	75	Plastic blister	NA	Ileum	CT	Open surgery	NA	(21)
2024	M	55	Fish bone	2.3	Jejunum	CT	Laparoscopy	5	(22)
2024	M	2	Magnetic beads	NA	Ileum, cecum, transverse colon	x-ray	Open surgery	10	(23)
2024	M	20	Toothpick	NA	Ileum	CT	Laparoscopy	5	(24)
2025	F	8	Magnet	0.2 × 0.3	Ileum, colon	x-ray, CT	Laparoscopy	9	(25)
2025	M	33	Fish bone	4.3	Ileocecal valve	x-ray, CT	Laparoscopy	7	(26)
2025	M	62	Plant stem	NA	Meckel diverticulum	Ultrasound	Laparoscopy	3	(27)
2025	F	51	Grill brush bristle	NA	Jejunum	CT	Laparoscopy	3	(28)
2025	M	40	Fish bone	4	Ileum	CT	Open surgery	27	(29)

## 4 Discusssion

About 80% of ingested foreign bodies pass through the gastrointestinal tract spontaneously and require no intervention. While around 20% of them need endoscopic evaluation for removal, and the remaining 1% demand surgical management (3). Sharp, hard, and elongated foreign bodies usually account for the latter two scenarios. They usually cause mucosal laceration and impaction in the gastrointestinal tract, and even penetrate the digestive wall, precipitating a cascade of complications including perforation, abscess, intestinal obstruction, aspiration, hemorrhage, and sepsis (26). These foreign bodies can be categorized into two types: non-metallic types (e.g., chicken bones, fish bones, and toothpicks) and metallic types (e.g., sewing needles, screws, coins, and magnets) (1, 3).

The type of foreign body ingestion differs significantly across all age groups. Infants, the elderly, patients with mental illness, and incarcerated individuals are high-risk groups. In infants, ingestion is mostly driven by inadequate supervision and innate curiosity, with common objects including coins, toys, batteries, and so on (1). Among the elderly, decreased masticatory function and loose or fallen dentures increase the risk of accidental ingestion of bones and dentures (30–33). For patients with mental illness and incarcerated individuals, foreign body inestion is mostly deliberate, mainly linked to self-mutilation or suicide attempts, and most ingested items are metallic (7, 34, 35). Compared with the high risk groups, adolescents and adults often experience food impaction due to pathological gastrointestinal strictures, tumors, rapid eating and other causes (3, 36, 37).

The foreign body obstruction and perforation most commonly occur at gastrointestinal sphincters, areas of narrowing, and acute bends. Among them, stomach (20%) and duodenum (23%) account for the most (2). Additional sites include appendix and sigmoid colon (4, 38), as well as regions of adhesion, diverticular disease and surgical anastomotic (27, 37–40). Notably, the perforation in our case was located in the ileum. We hypothesize the possible mechanisims as follows:the shape of the chicken bone fragment is small, which allowed it to traverse the pylorus and enter the small intestine; the small intestine is relatively narrow and long, which may increase the likelihood of sharp foreign bodies making contact, becoming impacted, and ultimately penetrating the intestinal wall; the rapid and powerful peristalsis of the small intestine leads to an unpredictable movement path of the chicken bone fragment, further predisposing to perforation.

Patients often ingest foreign bodies unintentionally and unknowingly. Furthermore, ingested foreign bodies may remain in the gastrointestinal tract for several days, weeks even years before the onset of obvious clinical symptoms, which not only delays diagnosis but also bring challenges for etiological investigation (6, 7). If foreign bodies are impacted in the esophagus, patients usually present with a foreign body sensation during swallowing, sometimes accompanied by odynophagia or retrosternal radiating pain (32). Once the foreign body traverses the esophagus into the gastrointestinal tract and cause, impaction, perforation or mechanical obstruction, patients may develop fever, persistent abdominal pain, tachycardia, and other clinical manifestations (26). Furthermore, the patients often present with abnormal inflammatory indicators, such as elevated peripheral white blood cell count, increased neutrophil ratio, and high C- reactive protein (41). As the condition progresses, patients may also present with hypotension, tachycardia, fever, and other related symptoms (5). However, the most symptoms and signs above are nonspecific, requiring imaging examination for diagnosis.

In our case, the patient presented with persistent abdominal pain as the chief complaint: the initial pain was a dull ache, which migrated and localized to the right lower abdomen a few hours later with marked tenderness at McBurney's point. Concurrently, the patient had an elevated peripheral white blood cell count. These clinical manifestations are somewhat consistent with the typical symptoms of acute appendicitis. However, it is also important to note that the patient had a definite history of foreign body ingestion, and combined with the imaging examination suggestive of a foreign body, the possibility of appendicitis can be clearly ruled out.

For high-density and radiopaque foreign bodies, such as coins and metal objects, x-ray can rapidly confirm the presence and location of the foreign body. It's easy to perform and can be used in regions that resource are limited. However, x-ray is not support to identify food boluses and other nonmetallic objects. In addition, although bones have high density, they are easy to be covered by fluid or soft tissues (1, 33, 42). In our case, the patient underwent x-ray examination, which failed to detect the foreign body. Nevertheless, based on the history of chicken bone ingestion, we proceeded to perform an abdominal CT scan.

Compared with x-ray, CT has a sensitivity of 100% and a specificity of 91%, making it the gold standard for diagnosis of foreign bodies investigation. It clearly show the size, shape, location and radiodensity of foreign bodies, and detects early signs of perforation, such as haziness of the local fat space, a small amount of free intraperitoneal fluid, and thickening of the intestinal wall (8, 9). In our case, CT not only identified the chicken bone and the precise site of perforation, but also revealed the fat space around the terminal ileum, mild peritoneal thickening, and small amount of free intraperitoneal fluid. It also excluded large abscesses, intestinal obstruction and other abdominal organs.

The treatment of foreign bodies ingestion depends on multiple factors, including the size and characteristics of the foreign body, the time and place they leave in the body and patient's clinical presentation. Even through approximately 80% of ingested foreign bodies will pass through the gastrointestinal tract spontaneously, close clinical observation is necessary in asymptomatic patients with foreign body ingestion (3). For foreign bodies confined to the upper gastrointestinal tract without intestinal wall penetration, endoscopic removal remains the optimal approach, with a success rate of more than 90% (3, 33, 43). When endoscopy fails to identify the foreign body, or when the patient has a definite history of foreign body ingestion or presents with related symptoms, imaging examinations and surgery should be performed immediately (6, 44).

Surgery should be considered over endoscopy removal in the following situations: the foreign body is sharp and hard, which may cause injury or perforation of surrounding tissues; the foreign body is large or irregular shaped; the foreign body is located beyond the safe distance of the endoscopy can reach; the patien's clinical status deteriorates or the foreign body has remained in the body for a long time (3, 5, 7, 26, 45). In our case, the chicken bone has caused perforation of the small intestine. Besides, due to the anatomical distance and the tortuous structure of the small intestine, endoscopy failed to visualize and remove the foreign body. Therefore, laparoscopic surgery was performed as the first-line approach to remove the foreign body.

Compared with traditional open surgery, laparoscopic surgery offers several advantages. It allows multi-angle observation, providing surgeons a clear and wide surgical field. Characterized by small incisions, minimal trauma, accurate manipulation, and reduced blood loss, it effectively alleviates postoperative pain, accelerates recovery, and shortens hospital stays. Additionally, It lowers the incidence of complications. The resulting scars are subtle, leading to a significantly better cosmetic outcome than open surgery (10–12).

In our case, the chicken bones was successfully removed from the patient by laparoscopic surgery. During laparoscopic exploration, the viability of the intestinal wall was carefully assessed. Although the surrounding mucosa appeared congested and edematous, no obvious intestinal necrosis, extensive tissue loss, or severe ischemia was identified. Moreover, the perforation was small (0.5 cm in diameter) and the abdominal contamination was mild and localized. Given these conditions, primary repair was considered safe, without the need for bowel resection. This approach also helps avoid potential complications related to intestinal anastomosis, and promote faster postoperative recovery.

The operative duration was appropriate. The patient had a good prognosis without any complications. However, it should be emphasized that laparoscopic surgery is not suitable for all patients. We still advice traditional open surgery for patients with large perforation, necrosis, severe contamination, or hemodynamic instability (14, 19–21, 29).

In summary, we report a 28-year-old male with small intestinal perforation caused by ingested chicken bone. Emergency laparoscopic surgery was performed successfully to extract the foreign body and repair the perforation. This case underscores the importance of maintaining clinical vigilance in patients with a history of foreign body ingestion, even when initial endoscopic studies are negative. It also highlights the efficacy of laparoscopic surgery for such intestinal perforation. Clinicians should remain cognizant of the potential complications associated with sharp foreign body ingestion and promptly utilize appropriate imaging studies and surgical intervention to ensure optimal patient outcomes.

## Data Availability

The original contributions presented in the study are included in the article/supplementary material, further inquiries can be directed to the corresponding author.
